# Supplementary material for: Characterisation of the British honey bee metagenome
Source: Nat Commun. 2018 Nov 26;9:4995. doi: 10.1038/s41467-018-07426-0 (PMC6255801; doi:10.1038/s41467-018-07426-0)
Supplement: Supplementary file 1 — Description of Additional Supplementary Files [file 41467_2018_7426_MOESM1_ESM.pdf]

Description of Additional Supplementary Files

File Name: Supplementary Data 1
Description: An analysis of completeness and contamination of all Metagenome Assembled Genomes (MAGs) following the MAGpy annotation pipeline (Stewart et al. 2018 DOI: 10.1038/s41467-018-03317-6).

File Name: Supplementary Data 2
Description: An analysis of completeness and contamination of all MCL clusters from the network analysis of contigs described in this study. Completeness and contamination were analysed following the MAGpy annotation pipeline (Stewart et al. 2018 DOI: 10.1038/s41467-018-03317-6).

File Name: Supplementary Software
Description: All scripts used in this study, including commands for each program used. This includes raw read QC, reference assembly, de novo contig assembly from non-mapping reads, Blobtools analysis, taxonomical assignation, BUSCO and MAGPy analysis.
