# Supplementary material for: Characterisation of the British honey bee metagenome
Source: Nat Commun. 2018 Nov 26;9:4995. doi: 10.1038/s41467-018-07426-0 (PMC6255801; doi:10.1038/s41467-018-07426-0)
Supplement: Supplementary file 3 — Supplementary Information [file 41467_2018_7426_MOESM3_ESM.pdf]

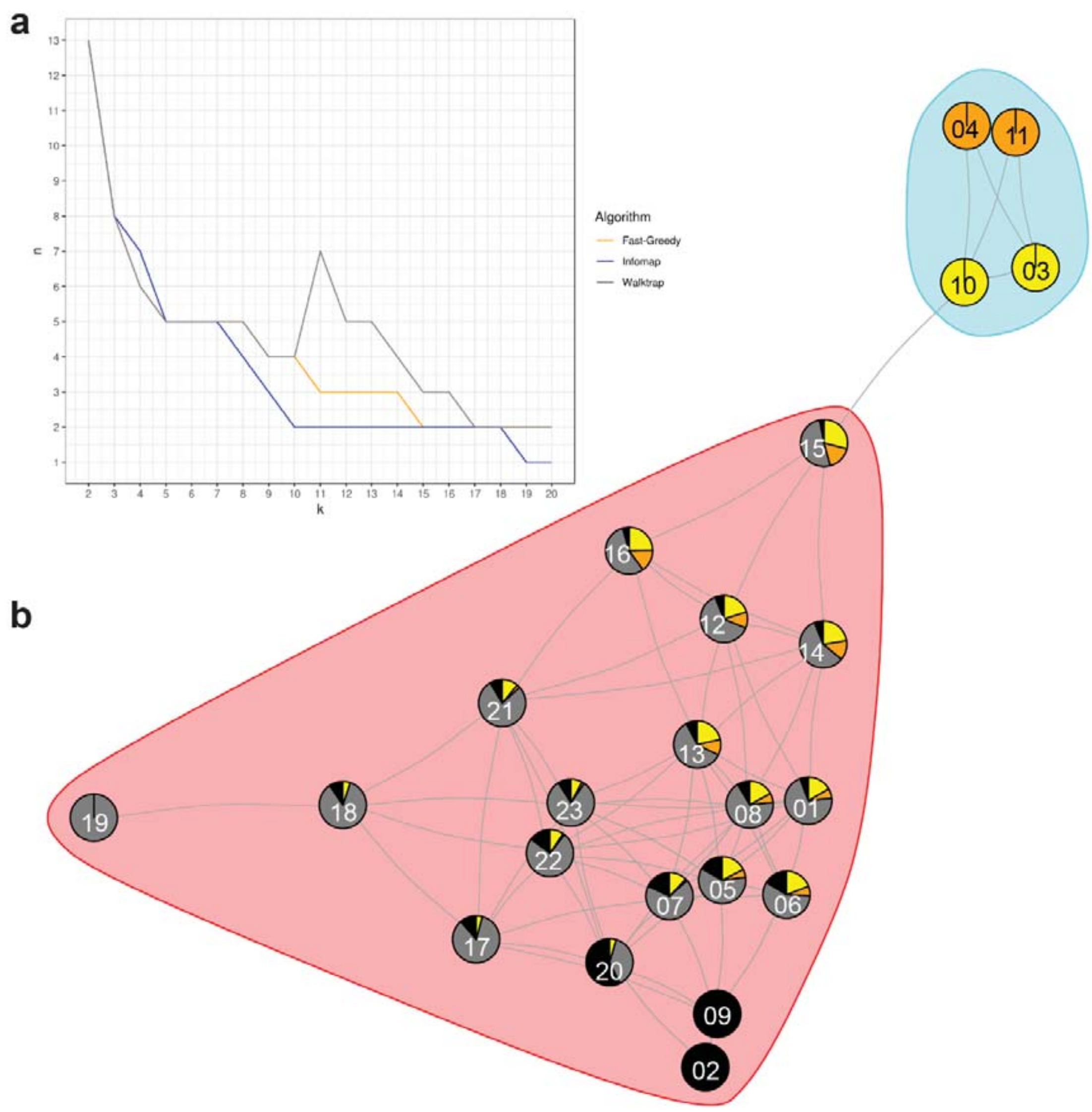

Supplementary Figure 1: Host population genetics analysis.
(a) k-Selection plot for k = 2 to 20 mutual k-nearest-neighbours and **(b)** accompanying network at k = 10, illustrating the 2 clusters (n) identified by the Infomap algorithm. Network nodes have been replaced with pie charts illustrating the ADMIXTURE results based on 4 genetic backgrounds.

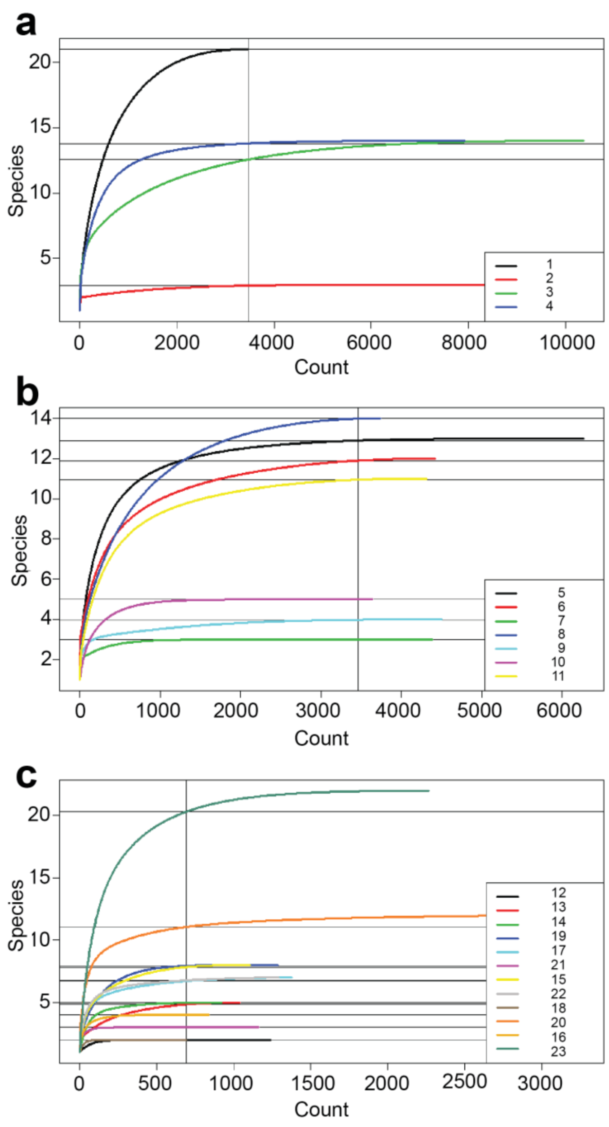

Supplemental Fig. 2: Rarefaction plot of micro-organisms identified. Mean species richness is displayed for all samples at each of the sequencing depths analysed: 50X reference genome coverage **(a)**, 25X reference genome coverage **(b)** and 17X reference genome coverage **(c)**. Each count on the x-axis represents a contig matching a unique species in the rDNA SILVA database while total number of species is displayed on the y-axis.

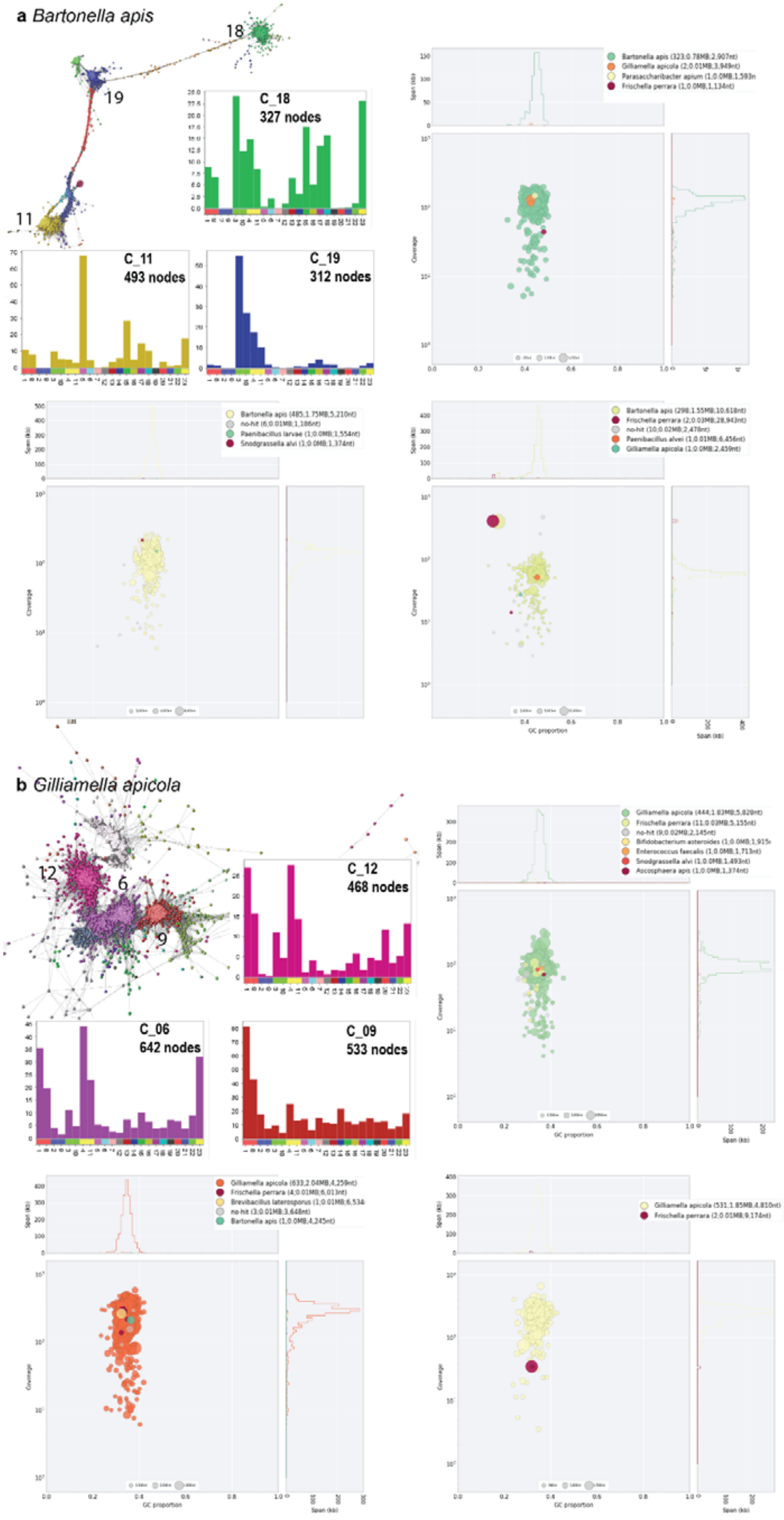
a Bartonella apis
18
19
11
C_18
327 nodes
C_11
493 nodes
C_19
312 nodes
Bartonella apis (323;0.78MB;2,907nt)
Gilliamella apicola (2;0.01MB;3,949nt)
Parasaccharibacter apium (1;0.0MB;1,593n
Frischella perrara (1;0.0MB;1,134nt)
Bartonella apis (485;1.75MB;5,210nt)
no-hit (6;0.01MB;1,186nt)
Paenibacillus larvae (1;0.0MB;1,554nt)
Snodgrassella alvi (1;0.0MB;1,374nt)
Bartonella apis (298;1.55MB;10,618nt)
Frischella perrara (2;0.03MB;28,943nt)
no-hit (10;0.02MB;2,478nt)
Paenibacillus alvei (1;0.01MB;6,456nt)
Gilliamella apicola (1;0.0MB;2,439nt)
GC proportion
Span (kb)
Coverage
b Gilliamella apicola
12
6
9
C_12
468 nodes
C_06
642 nodes
C_09
533 nodes
Gilliamella apicola (444;1.83MB;5,828nt)
Frischella perrara (11;0.03MB;5,155nt)
no-hit (9;0.02MB;2,145nt)
Bifidobacterium asteroides (1;0.0MB;1,915
Enterococcus faecalis (1;0.0MB;1,713nt)
Snodgrassella alvi (1;0.0MB;1,493nt)
Ascosphaera apis (1;0.0MB;1,374nt)
Gilliamella apicola (633;2.04MB;4,259nt)
Frischella perrara (4;0.01MB;6,013nt)
Brevibacillus laterosporus (1;0.01MB;6,534
no-hit (3;0.01MB;3,648nt)
Bartonella apis (1;0.0MB;4,245nt)
Gilliamella apicola (531;1.85MB;4,810nt)
Frischella perrara (2;0.01MB;9,174nt)

c *Lactobacillus*

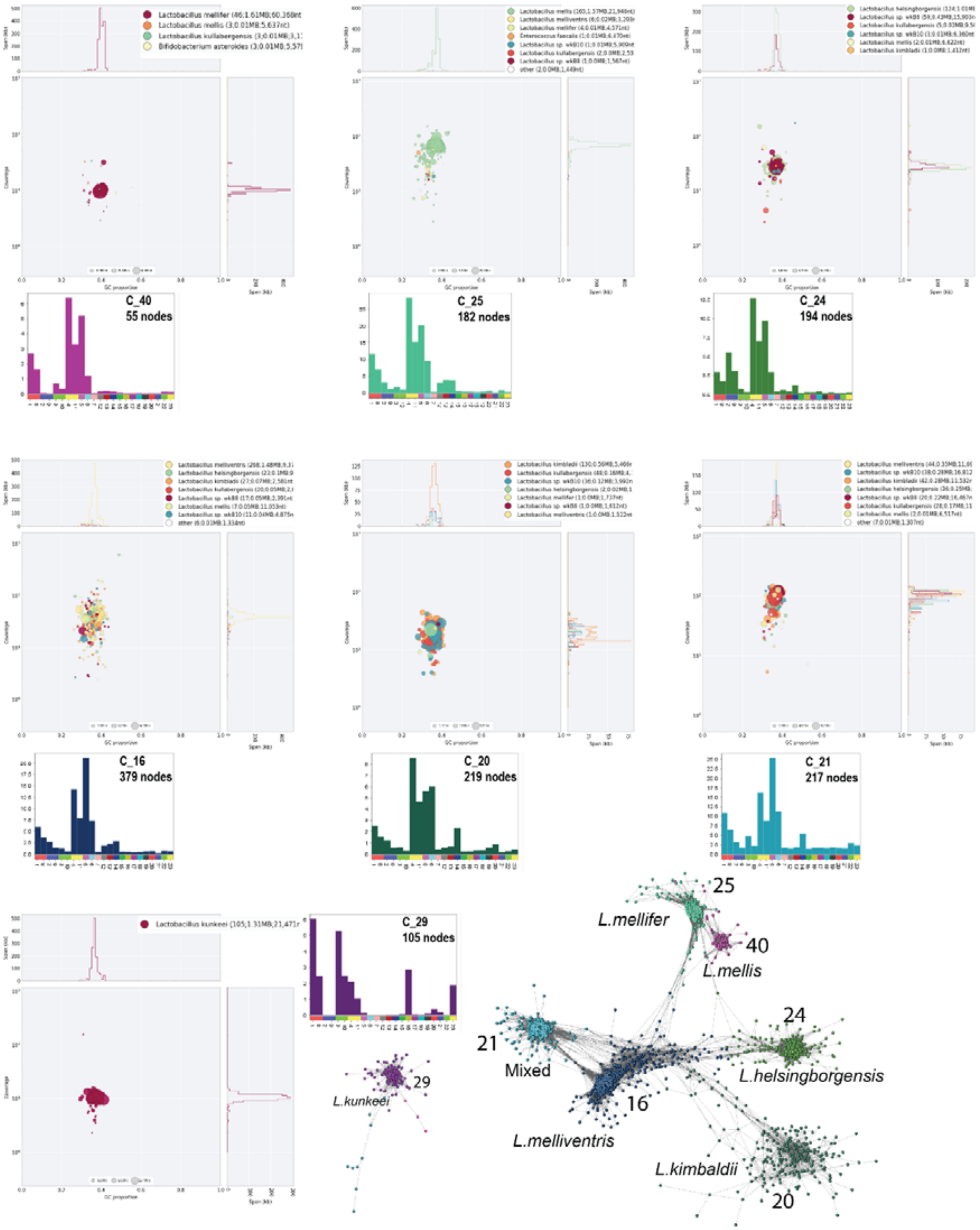

d Mixed taxa cluster

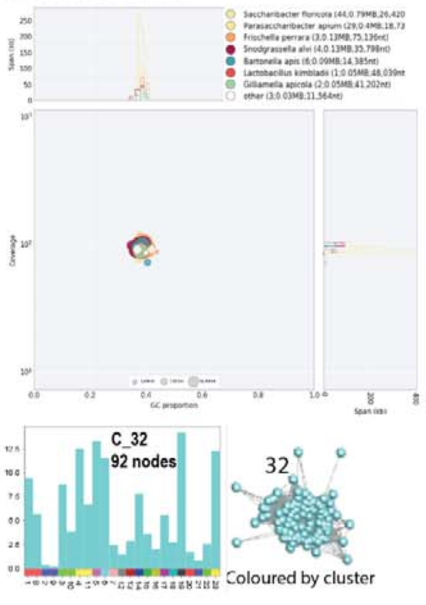

e Sample specific cluster

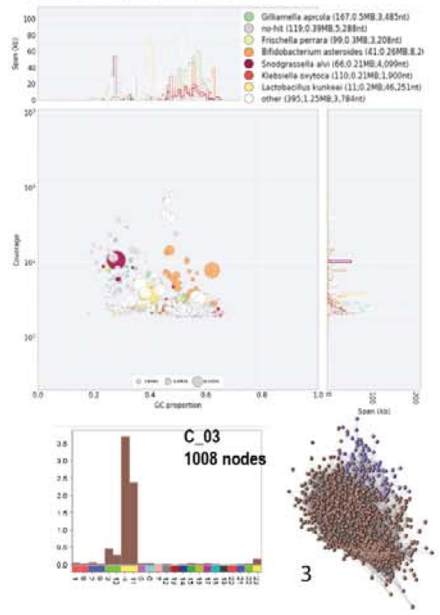

f

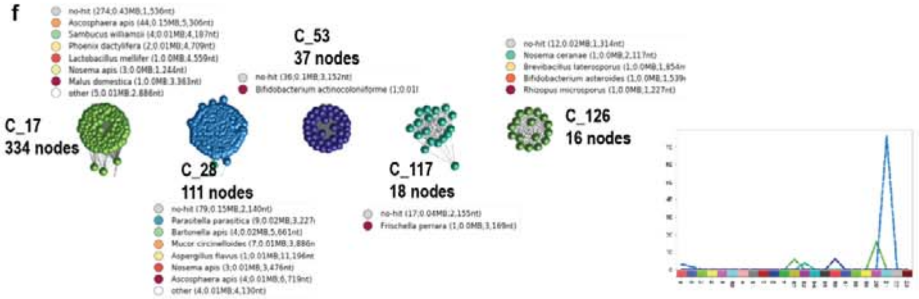

g Lotmaria passim

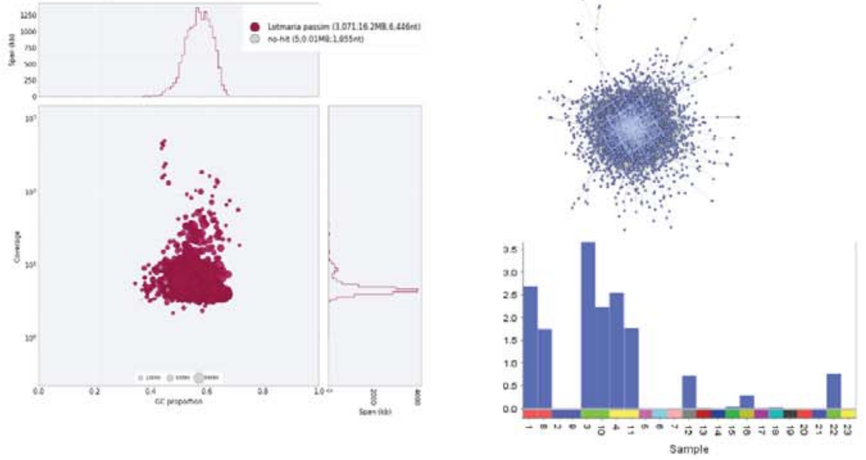

h Nosema apis

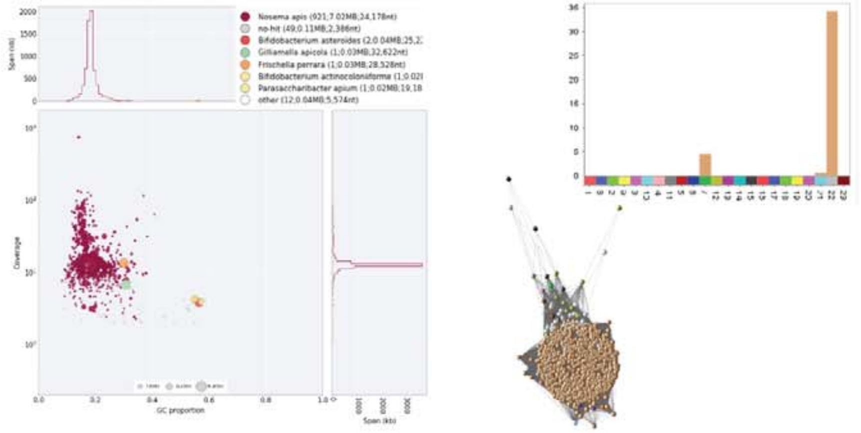

i Nosema ceranae

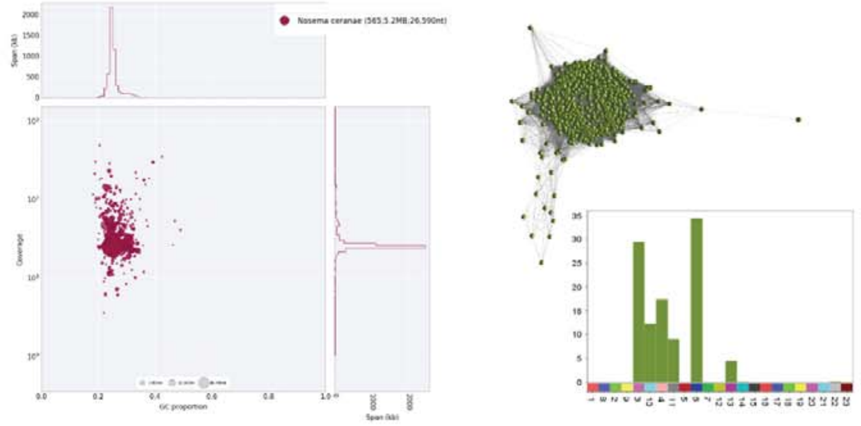

j Sample specific/Disease clusters

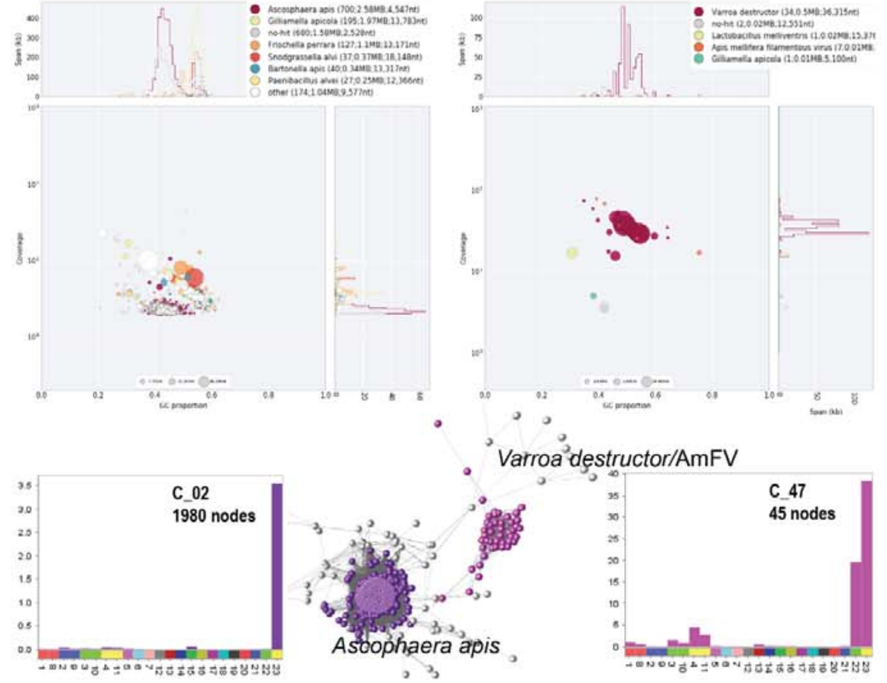

Supplementary Figure 3: Taxonomic summary of contig clusters

Clusters associated with honey bee cobionts including mean base coverage per contig (y axis) for each sample (x axis) and associated blobplot for **(a)** *Bartonella apis*, **(b)** *Gilliamella apicola* and **(c)** various *Lactobacillus* species, **(d)** a cluster of heterogeneous core taxa, **(e)** a cluster of contigs specific to sample 3, **(f)** clusters of contigs with poor annotation, **(g)** *Lotmaria passim*, **(h)** *Nosema apis*, **(i)** *Nosema ceranae* and **(j)** a community of species including *Ascophaera apis* (associated with chalkbrood), *Varroa destructor* and *Apis mellifera filamentous virus*.

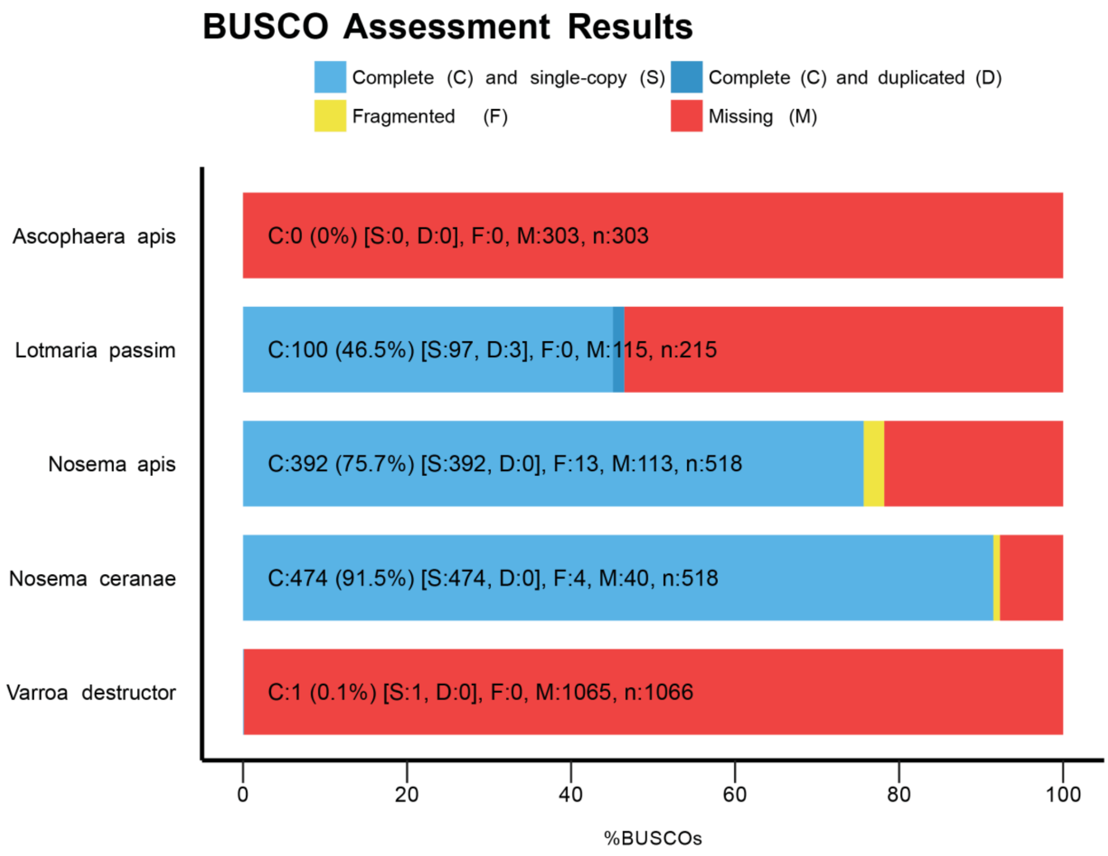

Supplementary Figure 4 Metagenomic binning analysis of Eukaryotic cobionts. Eukaryotic genome assembly and annotation completeness was analysed with Benchmarking Universal Single-Copy Orthologs (BUSCO) for the species indicated in the y-axis.

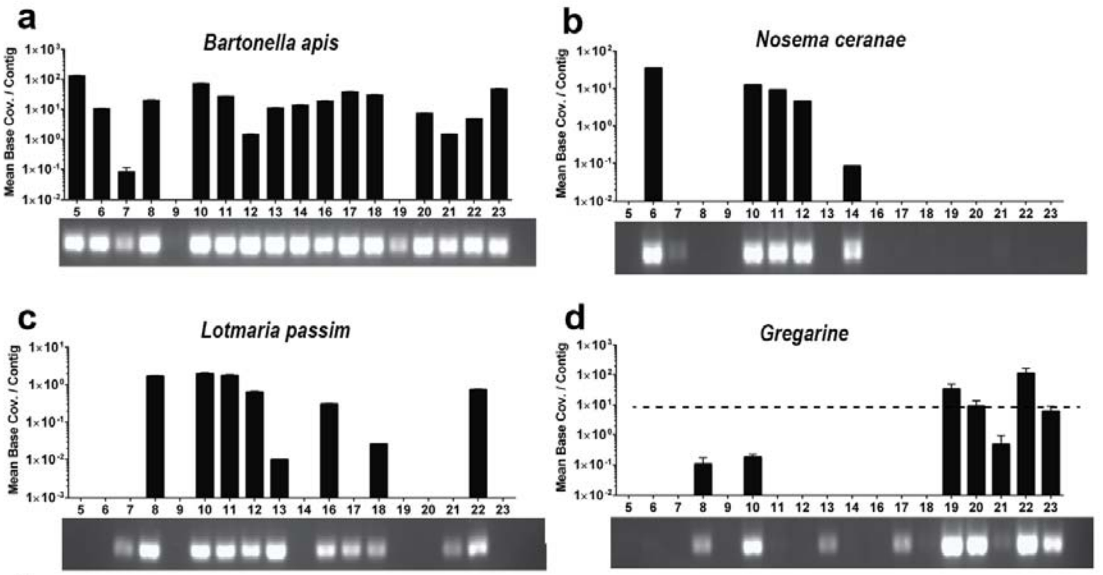

e

Description	Accession	Max score	Query cover	E value	Identity
**Gregarine contig**					
Ascogregarina taiwanensis 18S ribosomal RNA gene, partial sequence; and internal transcribed spacer 1, 5.8S ribosomal RNA gene, internal transcribed spacer 2, 26S ribosomal RNA and 5S ribosomal RNA genes, complete sequence	EF666482.1	1454	80%	0	88%
Neogregarinorida sp. OPPPC1 genes for 18S rRNA, ITS1, 5.8S rRNA, ITS2, 28S rRNA	AB748927.1	1378	75%	0	90%
Apicystis bombi partial ssu rRNA gene for small subunit ribosomal RNA, isolate Irw1	FN546182.1	2084	28%	0	100%
Apicystis sp. isolate Bpas1 18S small subunit ribosomal RNA gene, partial sequence	MF998086.1	1820	29%	0	95%
Mattesia sp. SV-2003 external transcribed spacer and 18S ribosomal RNA gene, partial sequence	AY334569.1	1796	30%	0	93%
**Gregarine rDNA**					
Apicystis bombi isolate NPaw1 small subunit ribosomal RNA gene, partial sequence	HQ619890.1	747	77%	0	99%
Apicystis bombi partial ssu rRNA gene for small subunit ribosomal RNA, isolate Irw1	FN546182.1	688	71%	0	99%
Apicystis bombi gene for small subunit ribosomal RNA, partial sequence	AB738024.1	686	70%	0	99%
Mattesia sp. SV-2003 external transcribed spacer and 18S ribosomal RNA gene, partial sequence	AY334569.1	656	86%	0	92%
Apicystis sp. isolate Bpas1 18S small subunit ribosomal RNA gene, partial sequence	MF998086.1	616	77%	3.00E-172	94%

Supplementary Figure 5: PCR based identification of known and putative *Apis mellifera* cobionts. Mean base coverage/contig (y axis) matched with the taxa indicated for each sample (x axis) against PCR bands for **(a)** *Bartonella apis*, **(b)** *Nosema ceranae,* **(c)** *Lotmaria passim* and an **(d)** a putative gregarine derived contig from Cluster 239 **(e)** BLAST results of PCR product sequences from (d) and rDNA.

Supplementary Table 1. Samples 1 to 4 were sequenced at depths of both 50 and 25 times reference genome coverage. All other samples were sequenced once at the depth indicated.

Sample ID	Vice county and postcode	Collection organized by	Coverage	% mapped to reference	Colony Information
1	Shropshire (SY8)	Roslin Institute	50X	89.4	A. m. mellifera breeding project
2	Fifeshire (KY13)	Roslin Institute	50X	97	A. m. mellifera (Colonsay Queen)
3	Ayrshire (KA23)	Roslin Institute	50X	92.1	Buckfast
4	Moray (IV36)	Roslin Institute	50X	91	Carniolan
5	Angus (DD3)	SASA	25X	88	Scottish population
6	West Lothian (EH48)	SASA	25X	93.3	Scottish population
7	Midlothian (EH25)	Roslin Institute	25X	95.9	Scottish population
8*	Shropshire (SY8)	Roslin Institute	25X	88.8	A. m. mellifera breeding project
9*	Fifeshire (KY13)	Roslin Institute	25X	97.3	A. m. mellifera (Colonsay Queen)
10*	Ayrshire (KA23)	Roslin Institute	25X	91.2	Buckfast
11*	Moray (IV36)	Roslin Institute	25X	89.9	Carniolan
12	Warwickshire	Fera	17X	91.1	A. m. mellifera (England)
13	Cambridgeshire	Fera	17X	93.7	A. m. mellifera (England)
14	Norfolk	Fera	17X	86.2	A. m. mellifera (England)
15	Wigtownshire (DG9)	SASA	17X	82.2	Scottish population
16	Banffshire (AB45)	SASA	17X	89.4	Scottish population
17	Argyllshire (PA38)	SASA	17X	90.5	Scottish population
18	East Ross and Cromarty (IV8)	SASA	17X	89	Scottish population
19	Mid Perthsire (PH6)	SASA	17X	88	Scottish population
20	Selkirkshire (TD7)	SASA	17X	92.1	Scottish population
21	East Perthshire (PH1)	SASA	17X	87	Scottish population
22	Lanarkshire (G74)	SASA	17X	87.2	Scottish population
23	West Inverness-shire (PH41)	SASA	17X	77.1	Scottish population

*Technical replicates of Samples 1-4.

Supplementary Table 2 The top 34 species ID hits, cumulative span, bit score and number of contigs (count) derived from *de novo* assembled non-reference matching reads.

Species	Phylum	Span	Score	Count
Gilliamella apicola	Proteobacteria	2.12E+07	2.15E+09	4828
Snodgrassella alvi	Proteobacteria	7.68E+06	1.29E+09	1816
Frischella perrara	Proteobacteria	8.18E+06	7.30E+08	1200
Bartonella apis	Proteobacteria	1.17E+07	6.23E+06	2694
Serratia fonticola	Proteobacteria	3.64E+05	4.18E+05	177
Klebsiella oxytoca	Proteobacteria	2.52E+05	3.57E+05	139
Parasaccharibacter apium	Proteobacteria	2.39E+06	1.89E+05	251
Hafnia alvei	Proteobacteria	1.77E+05	1.87E+05	65
Acinetobacter johnsonii	Proteobacteria	1.28E+05	1.50E+05	65
Klebsiella michiganensis	Proteobacteria	9.46E+04	1.13E+05	46
Saccharibacter floricola	Proteobacteria	2.73E+06	9.28E+04	208
Lotmaria passim	Trypanosomatida	2.40E+07	1.33E+08	6340
Lactobacillus kimbladii	Firmicutes	2.15E+06	4.16E+08	524
Lactobacillus kullabergensis	Firmicutes	1.67E+06	3.41E+08	503
Lactobacillus sp. wkB8	Firmicutes	9.47E+05	1.82E+08	183
Lactobacillus melliventris	Firmicutes	2.88E+06	1.37E+08	574
Lactobacillus helsingborgensis	Firmicutes	1.88E+06	1.14E+08	318
Lactobacillus kunkeei	Firmicutes	2.51E+06	1.09E+08	421
Lactobacillus mellis	Firmicutes	3.31E+06	1.07E+08	436
Lactobacillus mellifer	Firmicutes	2.33E+06	3.90E+07	176
Lactobacillus sp. wkB10	Firmicutes	1.26E+06	2.69E+07	346
Paenibacillus larvae	Firmicutes	6.21E+05	2.94E+06	127
Melissococcus plutonius	Firmicutes	3.71E+05	2.18E+06	25
Nosema apis	Microsporidia	7.05E+06	1.77E+07	932
Nosema ceranae	Microsporidia	5.30E+06	8.41E+06	598
Bifidobacterium asteroides	Actinobacteria	5.20E+06	8.72E+08	1057
Aspergillus flavus	Ascomycota	4.81E+04	6.32E+06	11
Ascosphaera apis	Ascomycota	2.92E+06	3.61E+05	805
Apis mellifera	Arthropoda	1.74E+05	5.48E+04	83
Apis cerana	Arthropoda	1.40E+05	3.61E+04	63
Varroa destructor	Arthropoda	5.37E+05	3.02E+04	51
Apis dorsata	Arthropoda	8.50E+04	2.04E+04	42
Apis florea	Arthropoda	8.06E+04	1.68E+04	35
Sambucus williamsii	Streptophyta	2.94E+04	3.70E+04	13
Trifolium repens	Streptophyta	3.26E+04	2.22E+04	19
Spiroplasma apis	Tenericutes	3.98E+04	1.76E+03	10
Apis mellifera filamentous virus	Viruses-undef	1.67E+04	1.40E+04	12
Apicystis bombi	Apicomplexa	8.33E+03	5.89E+02	3
no-hit*	no-hit	6.22E+06	0.00E+00	3264
undef*	undef	5.25E+06	5.44E+05	1864

*No-hit" = no match in our database. "Undef" = blast hits lacking "phylum" annotation.

Supplementary Table 3 Bioinformatics parameters used.

**Tool(s) used**	**Application**	**Parameters**	**Figures**
FastQC v0.11.2 [1]	Quality control of sequencing reads	See Supplementary File 1 for scripts or Github systems-immunology-roslin-institute/Honey-bee-metagenomics.	Fig. 1
Trimmomatic v0.35 [2]	Trimming reads of low quality regions and adapters	'Trailing:20 SlidingWindow:4:20 MinLen:100.	Fig. 1[2]
BWA-MEM v0.7.8 [3]	Reference assembly mapping of reads	-R and -M	Fig. 1
Picard Tools v2.1.1 [4]	Merging of read files and marking of duplicates	See Supplementary File 1 for scripts or Github systems-immunology-roslin-institute/Honey-bee-metagenomics.	Fig. 1
SAMtools view v1.3 [5]	Remove low quality alignments	-q 20	Fig. 1
GATK v3.5 Haplotype Caller [6]	Variant calling	--emitRefConfidence GVCF --variant_index_type LINEAR --variant_index_parameter 128000 -stand_emit_conf 30 -stand_call_conf 30	Fig. 1
GATK v3.5 VariantRecalibrator [6]	Variant quality score recalibration	-badLodCutoff -3 -an QD -an MQ -an MQRankSum -an ReadPosRankSum -an FS -an DP, dbSNP data as both the truth set (prior=15.0) and training set (prior=12.0)	Fig. 1
SNPeff v4.2 [7]	Identify SNVs affecting pro tein coding regions	See Supplementary File 1 for scripts or Github systems-immunology-roslin-institute/Honey-bee-metagenomics.	Fig. 1
SNPRelate [8]	Identity By State analysis based on SNVs across genome assemblies from all samples.	autosome.only = TRUE, remove.monosnp = TRUE, maf = NaN, missing.rate = NaN, verbose = TRUE	Fig. 1
SPAdes v3.8.1 [9]	*De novo* assembly of non-*Apis mellifera* mapping reads	--meta -t 96	Fig. 2, 3
NCBI BLASTN v2.6.0+ [10]	Assigning species identification to contigs from *de novo* assembly	-task megablast -outfmt '6 qseqid staxids bitscore std sscinames sskingdoms stitle' -culling_limit 5 -num_threads 32 -evalue 1e-25	Fig. 2, 3
Diamond v0.9.5 blastx [11]	Assigning species identification to contigs from *de novo* assembly	--max-hsps 1 --sensitive --evalue 1e-25 --max-target-seqs 1 --outfmt 6	Fig. 2, 3
Blobtools [12]	Species assignation and visualisation of contigs	See Supplementary File 1 for scripts or Github systems-immunology-roslin-institute/Honey-bee-metagenomics.	Fig. 2, 3
BUSCO [13]	Assessing eukaryotic genome assembly and annotation completeness.	-l eukaryota_odb9 -m geno	Supp. Fig. 4
MetaBat [14], CheckM [15], MAGpy [16]	Metagenomic binning analysis.	Followed pipeline from [17].	Supp. Data 1, 2

References

1. S. A. FastQC: a quality control tool for high throughput sequence data. Available online at: http://www.bioinformatics.babraham.ac.uk/projects/fastqc. (2010).

2. Bolger AM, Lohse M, Usadel B. Trimmomatic: a flexible trimmer for Illumina sequence data. *Bioinformatics* **30**, 2114-2120 (2014).

3. H. L. Aligning sequence reads, clone sequences and assembly contigs with BWA-MEM. *arXiv* **1303.3997v1**, (2013).

4. http://broadinstitute.github.io/picard/. (ed^(eds).

5. Li H*, et al.* The Sequence Alignment/Map format and SAMtools. *Bioinformatics* **25**, 2078-2079 (2009).

6. Van der Auwera GA*, et al.* From FastQ data to high confidence variant calls: the Genome Analysis Toolkit best practices pipeline. *Current protocols in bioinformatics* **43**, 11 10 11-33 (2013).

7. Cingolani P*, et al.* A program for annotating and predicting the effects of single nucleotide polymorphisms, SnpEff: SNPs in the genome of Drosophila melanogaster strain w1118; iso-2; iso-3. *Fly* **6**, 80-92 (2012).

8. Zheng X, Levine D, Shen J, Gogarten SM, Laurie C, Weir BS. A high-performance computing toolset for relatedness and principal component analysis of SNP data. *Bioinformatics* **28**, 3326-3328 (2012).

9. Bankevich A*, et al.* SPAdes: a new genome assembly algorithm and its applications to single-cell sequencing. *Journal of computational biology : a journal of computational molecular cell biology* **19**, 455-477 (2012).

10. Altschul SF, Gish W, Miller W, Myers EW, Lipman DJ. Basic local alignment search tool. *Journal of molecular biology* **215**, 403-410 (1990).

11. Buchfink B, Xie C, Huson DH. Fast and sensitive protein alignment using DIAMOND. *Nature methods* **12**, 59-60 (2015).

12. Laetsch D.R. BML. Interrogation of genome assemblies [version 1; referees: 2 approved with reservations]. *F1000Research* **6**, 1287 (2017).

13. Waterhouse RM*, et al.* BUSCO applications from quality assessments to gene prediction and phylogenomics. *Molecular biology and evolution*, (2017).

14. Kang DD, Froula J, Egan R, Wang Z. MetaBAT, an efficient tool for accurately reconstructing single genomes from complex microbial communities. *PeerJ* **3**, e1165 (2015).

15. Parks DH, Imelfort M, Skennerton CT, Hugenholtz P, Tyson GW. CheckM: assessing the quality of microbial genomes recovered from isolates, single cells, and metagenomes. *Genome research* **25**, 1043-1055 (2015).

16. Robert Stewart MA, Tim Snelling, Rainer Roehe, Mick Watson. MAGpy: a reproducible pipeline for the downstream analysis of metagenome-assembled genomes (MAGs). *bioRxiv*, (2017).

17. Stewart RD*, et al.* Assembly of 913 microbial genomes from metagenomic sequencing of the cow rumen. *Nature communications* **9**, 870 (2018).
