# Supplementary material for: Characterisation of the British honey bee metagenome
Source: Nat Commun. 2018 Nov 26;9:4995. doi: 10.1038/s41467-018-07426-0 (PMC6255801; doi:10.1038/s41467-018-07426-0)
Supplement: Supplementary file 7 — Supplementary Data 3 [file 41467_2018_7426_MOESM7_ESM.pdf]

Supplementary Data 3: ADMIXTURE efficacy testing.

Data for 39 worker bees from Harpur *et al.*[1] representing four lineages (A, C, M, Y) was downloaded from the European Nucleotide Archive (ENA; study accession: PRJNA216922), mapped to the honeybee reference genome (Amel4_5) and variants detected as described in Wragg *et al*.[2]. The resulting SNPs were filtered using Plink to remove (i) those not mapped to the autosomes, (ii) having low genotyping call rate (<0.9), (iii) low minor allele frequency (<0.1), and those with pairwise linkage disequilibrium r2 > 0.1 (for SNPs in 50 kb windows with a 10 kb step). The resulting 95,024 SNPs were submitted to unsupervised analysis in ADMIXTURE for $2 \leq K \leq 5$ genetic backgrounds, from which the CV error values (K = 2, CV = 0.84176; K = 3 CV = 0.70791; K = 4; CV = 0.63207; K = 5, CV = 0.84516) indicated K = 4 to be most likely, consistent with the number of lineages analysed and published analyses[1,2] **(Harpur 1)**.

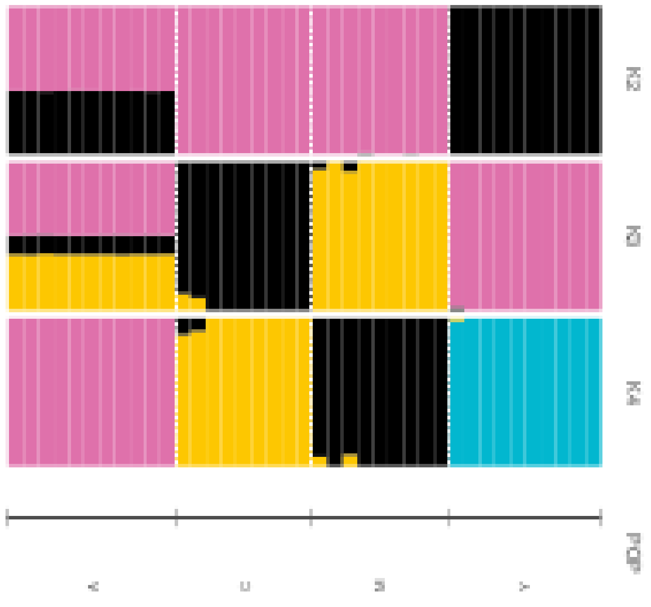

Harpur 1

For each population, the allele frequency for each SNP was used to simulate 16 genotypes assuming Hardy-Weinberg equilibrium, as described in the main text. The simulated genotypes from each population were merged and filtered as per the original dataset to remove those with low minor allele frequency (<0.1) and pairwise linkage disequilibrium r2 > 0.1 (for SNPs in 50 kb windows with a 10 kb step). The resulting 94,434 SNPs were submitted to unsupervised analysis in ADMIXTURE for $2 \leq K \leq 5$ genetic backgrounds, from which the CV error values (K = 2, CV = 0.7905; K = 3 CV = 0.59397; K = 4; CV = 0.49821;

K = 5, CV = 0.53711) indicated K = 4 to be most likely, consistent with the number of lineages analysed (**Harpur 2**).

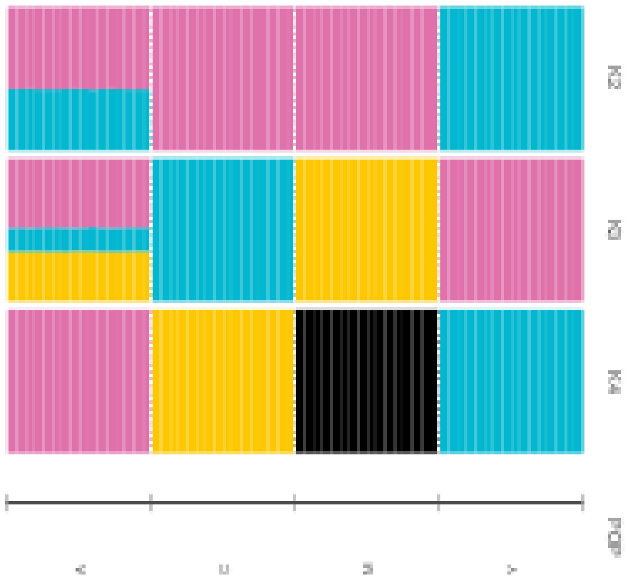

Harpur 2

References

1. Harpur, B. A. *et al.* Population genomics of the honey bee reveals strong signatures of positive selection on worker traits. *Proc. Natl. Acad. Sci.* **111**, 2614–2619 (2014).
2. Wragg, D. *et al.* Whole-genome resequencing of honeybee drones to detect genomic selection in a population managed for royal jelly. *Sci. Rep.* **6**, 27168 (2016).
